# Surface Architecture Influences the Rigidity of *Candida albicans* Cells

**DOI:** 10.3390/nano12030567

**Published:** 2022-02-07

**Authors:** Phuc H. Le, Duy H. K. Nguyen, Arturo Aburto Medina, Denver P. Linklater, Christian Loebbe, Russell J. Crawford, Shane MacLaughlin, Elena P. Ivanova

**Affiliations:** 1 STEM College, School of Science, RMIT University, Melbourne, VIC 3000, Australia; s3756681@student.rmit.edu.au (P.H.L.); nhkduy@gmail.com (D.H.K.N.); arturoaburto.medina@rmit.edu.au (A.A.M.); denver.linklater@rmit.edu.au (D.P.L.); russell.crawford@rmit.edu.au (R.J.C.); 2 ARC Research Hub for Australian Steel Manufacturing, STEM College, School of Science, RMIT University, Melbourne, VIC 3000, Australia; 3 SciTech Pty Ltd., Preston, VIC 3072, Australia; christian@scitech.com.au; 4 BlueScope Steel Ltd., Port Kembla, NSW 2505, Australia; Shane.Maclaughlin@bluescopesteel.com

**Keywords:** surface architecture, surface roughness, *Candida albicans*, biofilm formation, cell rigidity, Young’s modulus, atomic force microscopy

## Abstract

Atomic force microscopy (AFM) was used to investigate the morphology and rigidity of the opportunistic pathogenic yeast, *Candida albicans* ATCC 10231, during its attachment to surfaces of three levels of nanoscale surface roughness. Non-polished titanium (npTi), polished titanium (pTi), and glass with respective average surface roughness (*S_a_*) values of 389 nm, 14 nm, and 2 nm, kurtosis (*S*_kur_) values of 4, 16, and 4, and skewness (*S*_skw_) values of 1, 4, and 1 were used as representative examples of each type of nanoarchitecture. Thus, npTi and glass surfaces exhibited similar *S*_skw_ and *S*_kur_ values but highly disparate *S*_a_. *C. albicans* cells that had attached to the pTi surfaces exhibited a twofold increase in rigidity of 364 kPa compared to those yeast cells attached to the surfaces of npTi (164 kPa) and glass (185 kPa). The increased rigidity of the *C. albicans* cells on pTi was accompanied by a distinct round morphology, condensed F-actin distribution, lack of cortical actin patches, and the negligible production of cell-associated polymeric substances; however, an elevated production of loose extracellular polymeric substances (EPS) was observed. The differences in the physical response of *C. albicans* cells attached to the three surfaces suggested that the surface nanoarchitecture (characterized by skewness and kurtosis), rather than average surface roughness, could directly influence the rigidity of the *C. albicans* cells. This work contributes to the next-generation design of antifungal surfaces by exploiting *surface architecture* to control the extent of biofilm formation undertaken by yeast pathogens and highlights the importance of performing a detailed surface roughness characterization in order to identify and discriminate between the surface characteristics that may influence the extent of cell attachment and the subsequent behavior of the attached cells.

## 1. Introduction

*Candida albicans* biofilm formation is the leading cause of denture-related stomatitis and venous catheter infections [1]. If left untreated, the mortality rate of systemic candidiasis infection can be as high as 19–24% [2]. The ability of yeast cells and other microorganisms to adhere to different materials and types of surfaces enables biofilms to form on a wide array of medical devices and implantable materials, such as intravenous catheters, prosthetics, and joint replacements, as well as on different host tissues, resulting in persistent colonization and infections. It was previously reported that yeast cells have the ability to sense their physical surroundings, by a process known as contact sensing, to acquire information as to whether the surface is favorable for their virulence and/or attachment [3]. In the early stages of biofilm formation, planktonic cells attach to a surface followed by the formation of discrete colonies. These colonies then form a dense community of enclosed extracellular polymeric substances (EPS). During this intermediate phase, transformation of the *Candida* biofilm phenotypes occurs, from the usual round yeast shape to pseudohyphal or hyphal forms [1,4]. This transformation is thought to contribute to a range of virulence processes, including the ability to colonize abiotic and biotic surfaces (including human tissues), and the ability to form mature biofilms [5]. Maturation of the biofilm involves the development of a dense network of yeast, hyphae, and EPS. Finally, the biofilm-phenotype cells can release daughter cells to propagate the colonization and infection in other parts of the host.

To prevent surface colonization and biofilm formation, several surface modification approaches, including surface texturing, have been developed [6]. The impact of surface topography on cell orientation, proliferation, migration, differentiation, and mechano-sensing has also been a recent focus of research [7,8,9,10,11,12,13,14,15]. Several studies on mammalian and bacterial cells have demonstrated the relationship between surface topography and the physiological properties of the cells [15,16,17], particularly in light of the recent ability to design nanostructured surface topographies that exhibit biocidal properties towards attaching cells [18,19]. Cell rigidity is a distinctive mechanical property that measures the ability of a cell to resist the physical deformation caused by applied stress [20,21] and is attributed to the cell constituents of eukaryotic and prokaryotic cells, such as the cytoskeleton, cell wall, cell membrane, and capsule [21,22,23]. Since the cell wall acts as a barrier to protect cells from external physical stress, the rigidity of yeast cells is strongly associated with the cells’ responsiveness to such stimuli [24]. However, to the best of our knowledge, there is a lack of studies evaluating the relationship between the mechanical response (rigidity) of (attached) *C*. *albicans* cells and the surface nanoarchitecture. The investigation of the mechanical properties of cells (including their rigidity and shape) is important for further understanding the biophysical response of *C*. *albicans* cells during interaction with, and attachment to, abiotic surfaces.

In this study, we assessed the rigidity of *C. albicans* ATCC 10231 cells upon attachment to surfaces with various levels of nanoscale surface roughness, i.e., with decreasing average surface roughness (*S*_a_) values of 389, 14, and 2 nm, skewness (*S*_skw_) of 1, 4, and 1, and (*S*_kur_) of 4, 16, and 4, respectively, using non-polished titanium (npTi), polished titanium (pTi), and glass. Herein, the correlation between cell mechanical properties and the attachment propensity of *C. albicans* cells was studied using a combination of scanning electron microscopy, confocal scanning laser microscopy, and atomic force microscopy. The JPK NanoWizard Qi™ (JPK BioAFM Business, Bruker NanoGmbH, Berlin, Germany) mode was successfully employed to quantify the Young’s modulus of live *C. albicans* cell surfaces [25]. Given that the mechanical properties of *C. albicans* cells have a significant impact on their ability to proliferate, attach, and form biofilms on surfaces [26], the outcomes of this work provide useful insights into the design of antifungal surfaces.

## 2. Experimental

### 2.1. Surface Fabrication

Commercially pure Ti rods (ASTM Grade 2, purchased from Goodfellow, UK), 1 cm in diameter, were cut into discs of 1–1.5 mm in thickness using a Secotom-50 (Struers, Cleveland, OH, USA). The discs were separated into two groups. One group was directly used for experiments as npTi and the other group consisted of polished (pTi) samples. The polishing process was performed as described elsewhere [27]. The prepared Ti discs were sterilized by sequentially sonicating with MilliQ water (resistivity: 18.2 MΩ cm, 25 °C), 100% and 70% ethanol (EtOH) (Chem-Supply, Gillman, Australia) for 15 min each. The discs were then air-dried and kept in a desiccator prior to experiments.

Glass slides (ProSciTech, Kirwan, Australia) were cut into 1 × 1 cm^2^ pieces using a diamond pen (ProSciTech, Kirwan, Australia). The detailed cleaning steps and their purposes, using hydrochloric acid (Sigma-Aldrich Pty Ltd., Castle Hill, Australia) and a UV Ozone Cleaner (Ossila Ltd., Sheffield, UK), were described by Le et al. [27]. Glass surfaces were subjected to the same sterilizing and storing procedures as Ti surfaces before being used in attachment studies.

### 2.2. Scanning Electron Microscopy

An FEI Nova Nano Scanning Electron Microscope 200 FEGSEM (SEM) (FEI, Santa Barbara, CA, USA) mounted with Oxford X-Max20 Energy Dispersive X-Ray Detector (EDX) (Oxford Instruments, Abingdon, UK) was used to obtain typical surface micrographs and the survey elemental chart for all the sample surfaces at 3 kV under 1000× magnification in high vacuum condition.

### 2.3. Atomic Force Microscopy

A NanoWizard^®^4 scanning Atomic Force Microscope (AFM) (JPK BioAFM Business, Bruker NanoGmbH, Berlin, Germany) was used to visualize the surface topography of the substrata. The AFM head was mounted on an upright optical microscope (IX81, Olympus, Tokyo, Japan) and the experiments were carried out on an active vibration isolation table and in an acoustic hood (Accurion, Goettingen, Germany). The scans were performed in MilliQ water, at approximately 22 °C using an n-type antimony-doped silicon probe (SICON, AppNano, Mountain View, CA, USA) in JPK’s Quantitative Imaging™ (QI) mode. The spring constant of the cantilever was 0.1–0.6 N m^−1^. The thermal noise method to calibrate the cantilever’s spring constant was carried out in liquid condition. Scanning in QI mode was maintained at 2–4 nN with a corresponding Z-length of 1–2 µm for all samples. QI mode assisted in alleviating lateral forces and in gaining more control in vertical forces, compared to those forces in contact or tapping mode. A voltage ramp was applied to the z-actuator; thus, a complete force distance curve was recorded at each pixel in QI mode. The interactions of sample-tip were, therefore, obtained with high spatial resolution compared to classic force mapping or force volume mode (64 × 64 pixels or less). Triplicate scans were obtained for each surface in MilliQ water. A set of roughness parameters were then determined, including average roughness (*S*_a_), root mean square roughness (*S*_q_), skewness (*S*_skw_), kurtosis (*S*_kur_), and maximum height (*S*_max_) [28]. The surface scan areas were 10 µm × 10 µm, which were then analyzed using Gwyddion 2.53 software [29]. Amira software (Thermo Fisher Scientific, Waltham, MA, USA) was then employed to render the three-dimensional scans.

### 2.4. Surface Wettability

The static contact angles of water on the studied surfaces were measured using the sessile drop technique. The contact angle measurements were performed using a Phoenix-MT(T) instrument (SEO Co., Seoul, Korea). Surfaceware 9 software was then used to determine the water contact angle for each of the substrata. The results were an average of five separate measurements for each substratum sample.

### 2.5. X-ray Photoelectron Spectrometry

A Thermo Scientific K-alpha X-ray photoelectron spectrometer (XPS) (Thermo Fisher Scientific, Waltham, MA, USA) was employed to perform the elemental analysis of the substratum surfaces. The K-alpha XPS instrument was equipped with a monochromatic X-ray source (Al Kα, *h*ν = 1486.6 eV) operating at 150 W. Photoelectrons emitted at 90° to the surface from an area of 400 × 400 μm^2^ were analyzed at 200 eV for survey spectra and then at 50 eV for region spectra. Survey spectra were recorded at 1.0 eV per step, while the region spectra were taken at 0.1 eV per step. The relative atomic concentration of elements determined using XPS was quantified according to the peak area in the selected high-resolution region, with the appropriate sensitivity factors for the instrument being used. High-resolution scans were performed across each of the titanium 2p, carbon 1s, and oxygen 1s peaks.

### 2.6. Microorganism, Culture Conditions, and Sample Preparation

*Candida albicans* ATCC 10231 was purchased from American Type Culture Collection (ATCC, Manassas, VA, USA). The fungal stocks were prepared in 20% glycerol nutrient broth (Oxoid, Thermo Fisher Scientific Australia Pty Ltd, Scoresby, Australia) and stored at −80 °C.

Prior to each experiment, fungal cultures were refreshed from stock on potato dextrose agar (PDA) plates (Neogen^®^ Culture Media, Lansing, MI, USA) at 35 °C for 24 h. The working suspension was prepared by subculturing one colony from these plates into sterile potato dextrose (PDB) medium (Neogen^®^ Culture Media, Lansing, MI, USA), pH 7.2–7.4. Cell densities may vary; therefore, cell density was adjusted to OD_600 nm_ = 0.1 (approximately 10^6^ cells mL^−1^) to ensure a similar number of cells in each experiment.

Before incubation, nitrogen gas was used to flush any dust on sterile surface samples. Then, incubation of the fungal cultures was carried out as follows: npTi, pTi, and glass surfaces were placed in a Costar^®^ 12-well plate (Corning, Corning, NY, USA) and immersed in 3 mL of the previously prepared suspension of *C. albicans* cells. The samples were allowed to incubate at 35 °C for 3 days in duplicate. To prevent evaporation of the working suspension during a long-term incubation, Milli-Q^®^ (Merck Millipore, Burlington, MA, USA) water was filled up to unused wells to maintain a humid environment.

Before attachment studies, the surfaces were gently washed with 3 mL of MilliQ water in a new 12-well plate to remove any non-attached cells, then placed in a circular disk for subsequent confocal laser scanning microscopy (CLSM) and atomic force microscopy (AFM) analysis. The scans were performed in Milli-Q^®^ (Merck Millipore, Burlington, MA, USA) water to mimic the working suspension and to maintain the cell shape. Regarding SEM analysis, surfaces were fixed with 2.5% glutaraldehyde for an hour and then dehydrated with a series of ethanol (30%, 50%, 70 %, 90%, and 100% respectively) solutions for 15 min each. Three independent technical replicates were carried out. *C. albicans* grown on PDA plates was used as a positive control.

### 2.7. SEM Analysis

In all SEM experiments, the surfaces were coated with a 5 nm thick iridium film using an EM ACE600 sputter coater (Leica, Germany). High-resolution scanning electron micrographs were taken using an FEI Nova Nano Scanning Electron Microscope 200 FEGSEM at 3 kV under 6000× and 20,000× magnification in high vacuum condition. Image J 1.52a was used for image adjustment, including brightness, contrast, and scale bars [30]. Adobe Photoshop CS6 (Adobe Inc., San Jose, CA, USA) was used to highlight the attached cells.

### 2.8. Confocal Laser Scanning Microscopy (CLSM) Analysis

A Zeiss LSM 880 Airyscan upright CLSM (Carl Zeiss Microscopy, Jena, Germany) operated with a 63× water immersion objective (ZEISS 60× / 1.0 VIS-IR) was used for fluorescence imaging of *C. albicans* cells. The cells were stained with a Yeast Live-or-Dye™ Fixable Live/Dead staining kit (Biotium, Fremont, CA, USA) that includes Live-or-Dye™, a cell membrane-impermeable red dye with excitation/emission of 562/583 nm that covalently stains free amines on the intracellular proteins of dead cells, and Thiazole Orange, a cell membrane-permeable green dye with excitation/emission of 512/533 nm that stains the cytoplasm with nuclear accumulation of total cells. CF^™^ Dye Concanavalin A (Con A) (Biotium, Fremont, CA, USA), a blue fluorescent dye with excitation/emission of 360/430 nm that specifically binds toEPS components such as α-mannopyranosyl and α-glucopyranosyl, was used to visualize EPS production [31,32]. Phalloidin (Thermo Fisher Scientific, Suge Land, MA, USA), a green fluorescent dye with excitation/emission of 495/518 nm, was used to study the F-actin network. Results were derived from the average of at least three independent experiments, containing two replicates for each experiment.

### 2.9. Cell Topography

The JPK Nanowizard QI™ (JPK BioAFM Business, Bruker NanoGmbH, Berlin, Germany) mode was used to investigate the surface topography and rigidity of attached *C. albicans* cells on the three studied substrata, using an n-type antimony-doped silicon probe (SICON, AppNano, Billerica, CA, USA). The scans were performed under liquid conditions (Milli-Q^®^ (Merck Millipore, Burlington, MA, USA) H_2_O) at approximately 22 °C. Milli-Q^®^ water was used to prevent the formation of salt crystallization during the scan. The viability of yeast cells was not impacted when scanning in Milli-Q^®^ H_2_O [33]. The extend and retract speed of the cantilever was fixed at 100.0 µm s^−1^. The setpoint and Z-length were adjusted in the range of 2–4 nN and 5–7 µm, respectively, to adapt the shape of the *C. albicans* cells. The scan resolution was set at 256 × 256 pixels. The surface topography was analyzed using Gwyddion 2.53 software and visualized using Amira software for three-dimensional rendering.

### 2.10. Cell Rigidity

JPK SPM Data Processing software (JPK BioAFM Business, Bruker NanoGmbH, Berlin, Germany) was employed to analyze cell rigidity. Force-distance curves representing each pixel in a QI^™^ map (256 × 256 pixels) were processed by applying the Gaussian smoothing method with a width of 0.3 and the baseline adjustment with offset and tilt removal from a relative position of 200 nm to infinity. The height correction to determine the vertical position of the tip for cantilever bending and contact point adjustment (*x*-axis) were further utilized to standardize all force curves before the evaluation of Young’s modulus by fitting the approach data (extend line). The fit range on the approach curve was optimized from 0% to 23%. Data were then fitted using Hertz/Sneddon model with a quadratic pyramid tip shape (half-angle to edge) according to the following equation [34]:

$$E=\frac{\sqrt{2}}{tantan\;\alpha \;}\times \frac{F\left(1-\nu^{2}\right)}{\delta^{2}}$$

where E is the Young’s modulus representing rigidity, α is the edge angle of the silicon tip, F is the loading force, ν is Poisson’s ratio at 0.5 for soft biological samples, and δ is the indentation depth.

A total of 65,536 force-distance curves in each scan was analyzed using an automated batch fitting after importing the optimized processing method in JPKSPM software. The residual root mean square (RMS) error for elasticity fit was maintained below 150 pN. The processed QI data only at the central area of the cell body was used to eliminate the tip artifacts at the edge. Final quantification was conducted via the dataset extraction from the Young’s modulus map as integers by Gwyddion 2.53 software. These numbers were subsequently assembled into intervals for frequency histogram in Fiji-ImageJ 1.52b software [35]. These histograms were fitted with Gaussian function to observe nominal Young’s modulus values for specific materials. Results were derived from the average of at least three independent experiments, containing two replicates for each experiment.

### 2.11. Statistical Analysis

All data were expressed as means ± standard deviation, unless otherwise stated. Statistical data were analyzed by One-way ANOVA using IBM SPSS Statistics 26 software (IBM, Armonk, NY, USA). Values were considered statistically significant differences if *p*-values were less than 0.05 (* *p* < 0.05).

## 3. Results and Discussion

### 3.1. Surface Characterization

**Figure 1:**
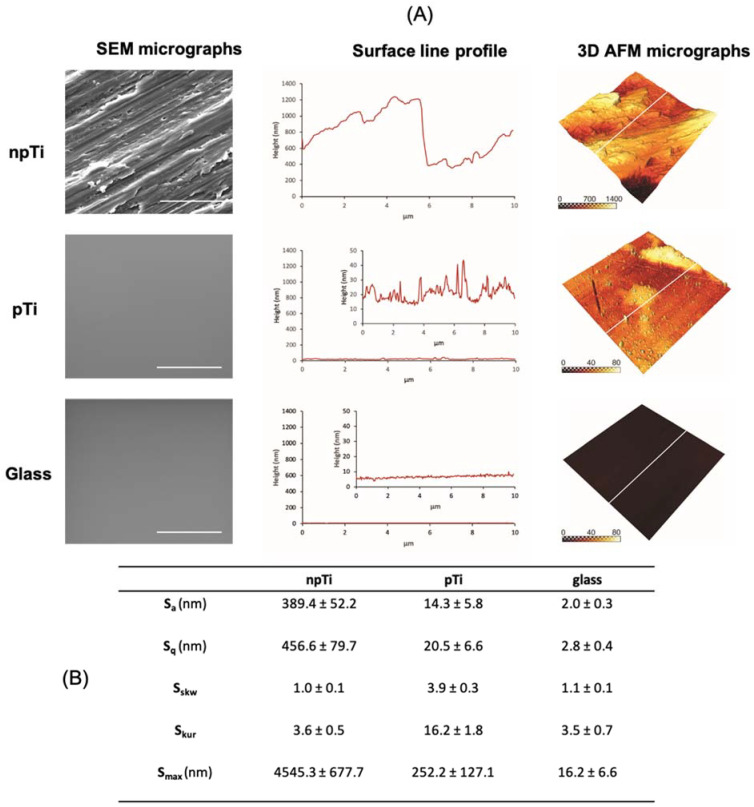
Surface topographic analysis of npTi, pTi, and glass surfaces. (**A**) SEM micrographs, AFM line profiles (scale bar = 50 µm), and corresponding 3D visualization of AFM micrographs showing the surface roughness of each respective sample. (**B**) Surface roughness parameters of the three studied surfaces as derived from 10 × 10 µm^2^ AFM scanning areas. The resolution of each scan was 256 × 256 pixels, n = 5.

In this study, we used surfaces with variable degree of nanoscale roughness. npTi, pTi, and glass surfaces were characterized by decreasing average surface roughness (*S*_a_) values of 389, 14, and 2 nm. The surface nanoarchitecture was further defined by two characteristic surface roughness parameters, kurtosis (*S*_kur_) and skewness (*S*_skw_). npTi, pTi, and glass surfaces exhibited *S*_kur_ values of 4, 16, and 4 and *S*_skw_ values of 1, 4, and 1, respectively, as inferred from the AFM topographic analysis (Figure 1). The *S*_skw_ values for all three surfaces studied were positive, indicating that the surfaces were composed mainly of peaks with a similar symmetry of height distribution. Two surfaces (npTi and glass), with a difference of two orders of magnitude in S*_a_* values, possessed similar *S*_kur_ values of 4, indicating that these surfaces had a similar proportion of spiky peaks and valleys. By contrast, an S_kur_ value of 16 measured for pTi surfaces was indicative of a surface with a higher number of peaks than valleys.

Along with the surface topographic analysis, surface wettability was confirmed by water contact angle (WCA) measurements. The WCA for npTi, pTi, and glass were 58.8°, 64.9°, and 5.3°, respectively (Table S1). Thus, both Ti surfaces (npTi and pTi) were slightly hydrophilic, whereas glass surfaces were superhydrophilic. According to EDS and XPS analysis, the surface chemistry of npTi and pTi was identical (Table S1 and Figure S1).

### 3.2. Cell Morphology of C. Albicans Attached on npTi, pTi, and Glass Surfaces

**Figure 2:**
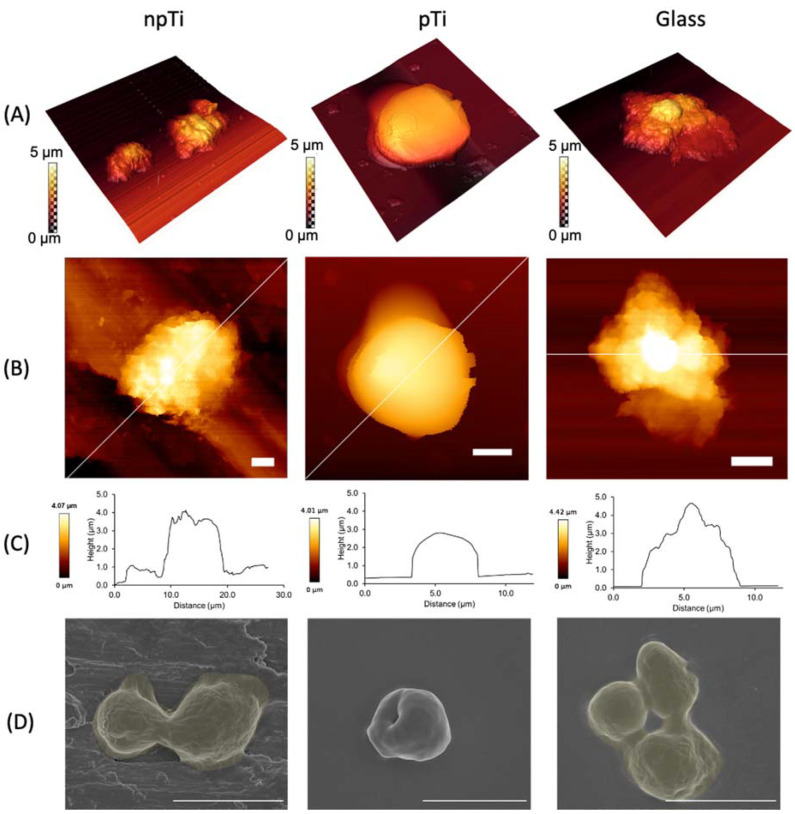
Morphology and topographic profile of *C. albicans* cells attached onto npTi, pTi, and glass surfaces. (**A**) 3D AFM micrographs of the surface morphology of *C. albicans* cells. (**B**) 2D AFM micrographs demonstrated the shape and orientation of cell attachment on different surfaces. Scale bars are 2 µm. (**C**) Corresponding surface line profiles revealed the dimension of *C. albicans* cells on npTi, pTi, and glass surfaces. (**D**) SEM micrographs showing the surface morphology of attached *C. albicans* cells after a 3-day incubation period on npTi, pTi, and glass surfaces. Rough EPS layers were detected on attached *C. albicans* cells on npTi and glass (false-colored yellow). Attached *C. albicans* cells on pTi were not covered by EPS. Scale bars are 5 µm.

*C. albicans* is a polymorphic fungus that exists either as a budding oval yeast (2–5 µm diameter) or as filamentous pseudohyphae or hyphae, with an average width of 2.8 and 2.0 µm, respectively [36]. Here, AFM was used to study the cell morphology upon attachment to npTi, pTi, and glass surfaces. *C. albicans* cells grown in PDB were used as the control. The diameter of the cells attached on npTi, pTi, and glass surfaces was measured to be 3.83 ± 0.63 µm, 3.56 ± 0.67 µm, and 3.78 ± 0.57 µm, respectively. The *C. albicans* cells attached on the surfaces after a 3-day incubation period appeared to be consistently smaller than the control cells (diameter of 5.83 ± 0.67 µm) (Supplementary Table S2). Although the cell height was determined to be approximately 3 µm on the three studied surfaces, atomic force microscopy scans revealed two distinct morphologies of *C. albicans* cells (Figure 2). On pTi surfaces, the cells exhibited a well-defined round shape and a smooth surface with S_a_ of 2.6 µm (Table 1). By contrast, the *C. albicans* cells attached on both glass and npTi surfaces possessed S_a_ = 3.3 µm and appeared elongated (non-spherical) in shape due to the production of cell-associated EPS.

Generally, there was a statistically significant difference in *S*_a_ between the central and peripheral parts of the *C. albicans* cells on pTi, compared to npTi and glass (Table 1). The dissimilarity in *S_a_* of the cell body could be explained by the alteration of cell surface topography during interaction with the surfaces and the heterogeneous buildup of EPS. Since *C. albicans* cells were grown and interacted with three studied surfaces under the same experimental conditions, it is suggested that the substratum surface characteristics might trigger the morphological changes to the *C. albicans* cells. In addition, these surface characteristics did not impact the viability of attached cells on the three different studied surfaces over a 5-day incubation period (Figure S2 and Figure S3).

### 3.3. Mechanical Properties and Biofilm Formation of Attached C. Albicans Cells on npTi, pTi, and Glass Surfaces

**Figure 3:**
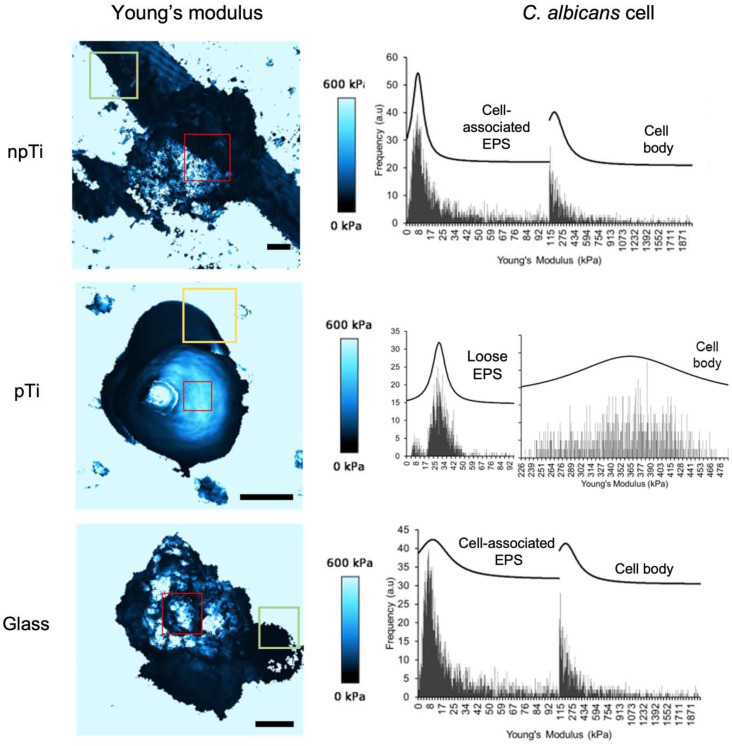
The mechanical properties of *C. albicans* cells. Representative Young’s modulus values of *C. albicans* cells attached on npTi, pTi, and glass surfaces after a 3-day incubation period. The elasticity of *C. albicans,* including cell body and excreted EPS, was obtained by using Hertz/Seddon model for soft materials on 65,536 AFM force curves. The dark and bright blue color in the elasticity maps represent soft and hard matter, respectively. Young’s modulus data highlighted by the red squares were extracted and constructed into histograms. These histograms were fitted with Gaussian function to extract a nominal Young’s modulus value (elasticity value) for specific materials, i.e., for the cell body and cell-associated EPS. Cell-associated EPS was not detected on *C. albicans* cells attached on pTi. The red, yellow, and green boxes indicate the cell body, the loose EPS, and the cell-associated EPS, respectively. Scale bars are 2 µm.

The difference in the cell morphology and surface roughness of *C. albicans* cells on the three studied surfaces raised the question as to whether the mechanical properties of the attached cells might be influenced by the surface topography and/or the architecture. In this context, the cell rigidity data that were generated using QI^™^ mode AFM (JPK BioAFM NanoGmbH, Berlin, Germany) were extracted from AFM maps and analyzed. The JPK NanoWizard^®^ QI^™^ (JPK BioAFM NanoGmbH, Berlin, Germany) mode movement algorithm eliminates lateral tip forces and controls vertical forces during scanning, allowing full control over the tip-sample interactions at every pixel [37]. Based on the force-distance curves, a more comprehensive and quantitative set of data for the cell mechanical properties can be extracted. The relative rigidity (expressed in kPa) of the *C. albicans* cells is shown in Table 1 and Figure 3. The rigidity of *C. albicans* ATCC 10231 cells grown in PDB (used as a control) was found to be in the range of 150–190 kPa, which is in agreement with the rigidity values reported by other groups [38,39,40]. The Young’s modulus *E* values were arranged into frequency histograms for the estimation of cell rigidity of *C. albicans* cells attached on both npTi and glass surfaces. Two statistically meaningful distributions in cell rigidity were observed, representing the stiffness of cell-associated EPS at 8 kPa–10 kPa and the cell body at 164–185 kPa. Loose EPS (not cell-associated) with similar stiffness values of 9 and 2 kPa was observed on both the npTi and glass substrata. The presence of EPS is most evident in the elasticity maps presented in Figure 3. Negligible production of cell-associated EPS was detected on the surface of *C. albicans* cells attached on pTi surfaces; hence, the cell rigidity values were consistently measured to be 364 ± 3 kPa, a statistically significant twofold increase compared to those cells attached on npTi and glass surfaces. The lack of cell-associated EPS production for *C. albicans* cells attached to pTi surfaces was further evidenced by SEM imaging (Figure 2D and Figure S4). Any loose EPS that were detected surrounding the *C. albicans* cells on pTi exhibited greater stiffness of 29 kPa (Figure 3). Furthermore, an elevated amount of loosely associated EPS was detected on npTi and glass surfaces, most likely in order to facilitate the cells’ attachment [41].

The presence of budding (asexual reproduction by an asymmetric division [38]) for most of the *C. albicans* cells, regardless of the substrata, was evidenced by examining SEM micrographs (Figure 2D). The rigidity of yeast-budding was also studied and analyzed using AFM (Figure S5 and Figure S6). The rigidity of the yeast cell bud on pTi surfaces was measured to be 793 ± 19 kPa.

**Figure 4:**
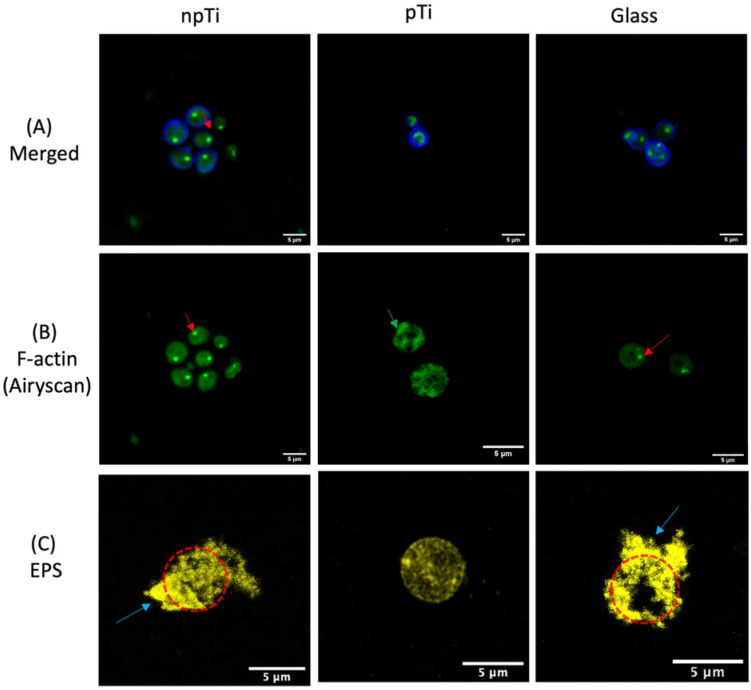
Cell wall, F-actin staining, and EPS production of *C. albicans* attached on npTi, pTi, and glass surfaces after a 3-day incubation period. (**A**) CLSM micrographs of *C. albicans* cells stained with calcofluor white (blue color) and phalloidin, which selectively stains F-actin (green color). (**B**) High-resolution CLSM (Airyscan) micrographs of filamentous actin distribution of *C. albicans* cells on the three studied surfaces. The red arrow indicates representative actin patches. The green arrow shows the lack of actin patches and the condenses in F-actin distribution of attached cells on pTi. (**C**) *C. albicans* cells were stained with Concanavalin A, which specifically binds to the a-mannopyranosyl and a-glucopyranosyl glycoprotein components of EPS (yellow). The EPS formed by the *C. albicans* was developed on npTi and glass surfaces after a 3-day incubation period. The blue arrow indicates the EPS surrounding attached cells on npTi and glass. The cell body was detected underneath the EPS, indicated by the red dashed circle. By contrast, attached cells on pTi were not enclosed by any EPS. Scale bars are 5 µm.

The detection of EPS of attached cells on the studied surfaces was conducted using CLSM. The *C. albicans* cells attached on npTi and glass surfaces were surrounded by EPS layers. In contrast, most of the *C. albicans* cells attached on pTi were not enveloped by any EPS layers (Figure 4C).

### 3.4. Physiological Properties of C. Albicans Cells Attached on npTi, pTi, and Glass Surfaces

To obtain an in-depth understanding of the physiological response of the *C. albicans* cells attached to the surfaces with different architecture, the cell cytoskeleton of the attached *C. albicans* cells was visualized via fluorescence staining of F-actin. The actin cytoskeleton is instrumental in many crucial cellular processes, including morphogenesis process and hyphal formation [42,43]. Its components mainly consist of cortical actin patches located at polarized growth spots, and actin filaments that act as tracks for vesicle secretion to the polarized growth sites [44]. Here, it was found that the F-actin distributions of *C. albicans* cells attached on pTi were more condensed, in comparison to those cells observed on npTi and glass surfaces. Moreover, cortical actin patches were absent in *C. albicans* cells on pTi surfaces, whereas the actin patches in *C. albicans* cells on the other studied surfaces were clearly observed (Figure 4). Pathogenic yeast *C. albicans* is competent in responding to a range of environmental stresses. Yeast cells can sense a certain stressor and transfer these signals through mitogen-activated protein kinase (MAPK) modules to trigger the stress response pathway [45]. These environmental stresses might be involved in the damage of DNA, the cell wall, and, especially, the actin cytoskeleton. In this case, the actin cytoskeleton is required to re-polarize as a recovery response to continue the cell division process [46]. It is likely that, here, the condensed F-actin filaments and the lack of cortical actin patches of *C. albicans* cells on pTi may explain the significantly low cell attachment (Figure S2), and the lack of morphological transformations from round shape to pseudohyphae, or elongated hyphae on pTi, as reported in elsewhere [27].

## 4. Discussion

Yeast cell rigidity is strongly associated with yeast cellular structure [47], which can be modified in response to external stress [48,49,50,51,52]. *C. albicans* cells have been reported to increase their cell rigidity in response to various factors, such as the alteration of the nutrient medium [53,54], exposure to ethanol [48], a compromised or removed mannan layer in the cell wall [47], or the echinocandins-induced inhibition of β-1,3-glucan synthase, an enzyme involved in cell wall synthesis [53]. For example, *C. lusitaniae* cells were reported to exhibit increased levels of rigidity, with Young’s modulus values ranging from ~1250–1800 kPa, in cells treated with the antifungal agent, caspofungin (which induces a decrease of β-glucan and an increase in chitin production) [49]. Similarly, the cell mechanical properties of *S. cerevisiae* BY4741 and *C. albicans* (ABC Platform Bugs Bank, Nancy, France) cells also changed after treatment with caspofungin, with the cell rigidity of *S. cerevisiae* cells increasing from 529 ± 265 kPa to 1125 ± 468 kPa and the cell rigidity of *C. albicans* cells increasing from 186 ± 89 kPa to 1326 ± 340 kPa [38]. Notably, there is some inconsistency in the reported values of the cell rigidity of the *C. albicans* cells, ranging from 90–733 kPa. It is likely that these differences are strain- and growth-condition dependent [38,39,50,51,52].

It has been also shown that surface topography impacts the rigidity of eukaryotic cells [55]. For example, Yang et al. demonstrated that wrinkled polydimethylsiloxane surfaces with a wavelength of 2.5 µm and an amplitude of 0.75 µm could stimulate human bone marrow derived-mesenchymal stem cells (MSCs) to become more stiff [55]. The Young’s modulus of stem cells (4 kPa) was measured on flat surfaces, compared to 6 kPa on surfaces with a wrinkled topography after a 14-day incubation period [55]. However, similar h-MSCs were shown to exhibit a decrease in cell rigidity when attached to 350 nm grating-nanopatterned polystyrene surfaces, compared to measurements on flat control surfaces. Thus, the change in cell rigidity might result from a complex interplay between the nanotopography, stiffness and chemistry of the substrates surface.

**Figure 5:**
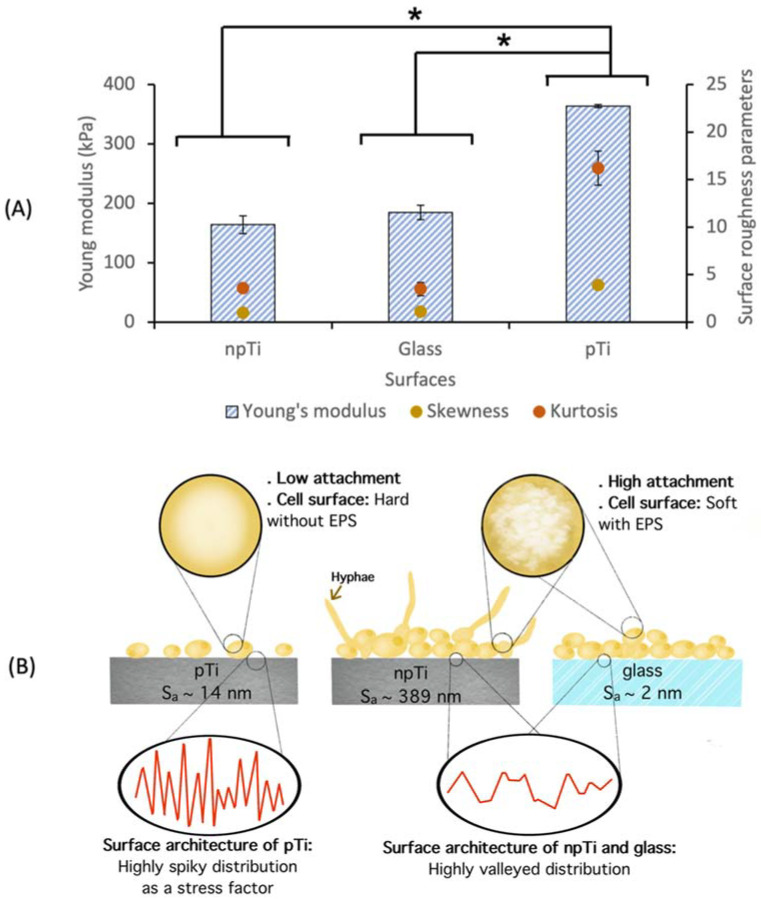
Correlation between surface roughness parameters (right *y*-axis) and mechanical properties of *C. albicans* cells (left *y*-axis). (**A**) The rigidity of *C. albicans* cells attached on npTi, glass, and pTi surfaces was analyzed after a 3-day incubation period. The values of the two surface roughness parameters, such as skewness and kurtosis, highlight the differences in surface architecture. Skewness and kurtosis reflect the proportion of valleys or peaks and the proportion of high peaks and hollow valleys, respectively. An increased cell rigidity was observed on surfaces with increased kurtosis values. A statistically significant difference in surface roughness parameters and Young’s modulus of attached *C. albicans* cells on pTi surfaces, compared to those on Ti and glass surfaces, is indicated by the asterisks (* *p* < 0.05). (**B**) Comparison of mechanical properties of *C. albicans* cells when settling on surfaces of varied in roughness and architecture. Hyphae were formed after 7-day incubation with npTi surfaces, as reported in a previous work [27].

The results obtained in this work indicated that a nanostructured surface with a characteristic surface architecture possessing a higher ratio of peaks than valleys prompted the increased rigidity of *C. albicans* cells attached to the surface (Figure 5), which was further associated with a chain of other cell phenotypic responses. Specifically, the cells were reduced in size and exhibited a round morphology. They exhibited condensed F-actin distributions and a lack of cortical actin patches. Additionally, they produced a negligible volume of cell-associated polymeric substances; however, they excreted a greater amount of loosely associated EPS. These results suggest that the change in cell rigidity is a response to a surface architecture that is unfavorable for the attachment of *C. albicans* cells. It seems that despite the greater production of loose EPS, cells are unable to attach and colonize the surfaces of pTi. In our previous study, we showed that the surface topography and architecture of pTi did not impact the viability of *C. albicans* cells but could inhibit their attachment propensity and the extent of biofilm formation, in which the number of *C. albicans* attached cells on pTi was threefold lower than the number of attached cells on npTi and glass [27].

Current research has consistently shown that the surfaces of bulk materials compared to those of their nanostructured topography counterparts can indeed affect the extent of attachment and biofilm formation of *C. albicans* cells, regardless of the substrate material. For example, it was shown that a surface topography exhibiting a square pattern of 120 nm diameter pits on medically relevant polymers reduced the number of attaching *C. albicans*, compared to the number of attached cells on the control smooth surfaces [56]. Additionally, titania nanoparticle coatings were shown to decrease the attachment and proliferation of *C. albicans* cells, whereas a planar TiO_2_ coating did not result in the same effect [57]. It was also reported that nanostructured surfaces showed a significant reduction in the attachment of the yeast-like fungi, *S. cerevisiae* and *C. albicans*, compared to the results obtained on non-structured surfaces [58,59]. Nowlin et al. also emphasized that the nanotopography of insect wing surfaces strongly impacted the extent of attachment of *S. cerevisiae* cells [58]. Similarly, Nana and Dennis observed that the nanostructured surface of the wings of *Neotibicen tibicen* cicadas possessed a spiky surface profile that could prevent the attachment and the hyphae morphogenesis of *C. albicans* cells [59].

We hypothesize that a nanoscale surface architecture of a substrate may be used to delay the pathogenicity of attached *C. albicans* cells. Indeed, the cell wall serves as a barrier to protect the cells from any external physical stresses, and yeast cell rigidity is significantly correlated with cells’ ability to respond to such stimuli [24]. Modifications to the cell wall, such as increases in cell rigidity, are ultimately accountable for a particular cell morphology [60]. Thus, it is believed that an increase in the rigidity of *C. albicans* cells attached onto pTi surfaces represents the cells’ response to the external stress factor provided by the unfavorable nanoscale surface architecture of the substrate to which they are attached. Recently, it was shown that *C. albicans* cells were unable to form hyphae and biofilms after seven days of interaction with pTi surfaces [27].

## 5. Conclusions

In summary, it was found that the rigidity of *C. albicans* cells was significantly higher when attached to pTi surface (363.8 kPa) in comparison to those cells on npTi (164.2 kPa) and glass (184.6 kPa). We also found a correlation between the surface architecture parameters and the rigidity of *Candida* cells. Specifically, nanoscale surface architectures that possessed a higher ratio of peaks than valleys, such as those observed on pTi surfaces, could not only resist *C. albicans* attachment but also act as an external stress factor, inducing an altered actin cytoskeleton network and yeast cells that attached. By contrast, surfaces that possessed a nanoscale valley-like architecture were more favorable towards the attachment and growth of *C. albicans* cells. The results of this study showed that surface architecture may affect the mechanical properties of *C. albicans* cells, although further studies toward understanding the metabolic pathways that regulate the modulation of the rigidity of the cells during attachment to substrate surfaces are required in order to obtain a deeper understanding of this phenomenon. Since the rigidity of yeast cells is a unique mechanical characteristic that quantifies a cell’s capacity to withstand physical deformation and is strongly associated with biofilm development [60], the correlative analysis of the surface nanoarchitecture and the mechanical properties of *C. albicans* cells could greatly benefit the design and development of new antifungal surfaces.

## Figures and Tables

**Table 1:** Analysis of *C. albicans* cell mechanical properties on npTi, pTi, and glass surfaces ^a^.

		npTi	pTi	Glass
*C. albicans* Cell Surface	S_a_ (µm)	3.3 ± 0.2	2.6 ± 0.1 *	3.3 ± 0.2
	Cell body (kPa)	164.2 ± 15.0	363.8 ± 2.5 *	184.6 ± 12.0
	Cell-associated EPS (kPa)	7.8 ± 0.1	*Not detected*	10.3 ± 0.7
	Loose EPS (kPa) ^b^	9.1 ± 0.1	29.0 ± 0.2	2.2 ± 0.1

^a^ Young’s modulus values were collected using AFM of *C. albicans* cells incubated on the three studied surfaces for 3 days. ^b^ “Loose EPS” refers to any EPS that is not associated with cells. * Surface roughness and rigidity of the attached *C. albicans* cell body has statistically significant difference on pTi surfaces compared to npTi and glass surfaces; *p* < 0.05.

## Data Availability

The data obtained during the course of this study has been stored in accordance with the policy pertaining to RMIT University requirements for PhD candidates.

## Supplementary Materials

The following supporting information can be downloaded at: https://www.mdpi.com/article/10.3390/nano12030567/s1, Table S1: Elemental composition and wettability of Ti, pTi, and glass surfaces; Table S2: The size of *C. albicans* cells from suspension and attached on npTi, pTi, and glass; Figure S1: The chemical composition of npTi, pTi, and glass surfaces. XPS-spectra of npTi, pTi, and glass surfaces (left) and correlating narrow scan spectra of Ti2p and Si2p positions (right); Figure S2: Representative SEM micrographs of *C. albicans* cells attached on npTi, pTi, and glass over 3-day incubation period; Figure S3: The morphology, viability, and attachment patterns of *C. albicans* cells on npTi, pTi, and glass surfaces; Figure S4: AFM micrographs showing the shape and orientation of cell attachment on npTi, pTi, and glass; Figure S5: The mechanical properties of *C. albicans* budding; Figure S6: Representative force-distance curves in QI data of *C. albicans* cells attached on Ti, glass and pTi surfaces after a 3-day incubation period.

## References

[1] Kojic E.M., Darouiche R.O. (2004). *Candida* Infections of Medical Devices. Clin. Microbiol. Rev..

[2] Magill S.S., Edwards J.R., Bamberg W., Beldavs Z.G., Dumyati G., Kainer M.A., Lynfield R., Maloney M., McAllister-Hollod L., Nadle J. (2014). Multistate point-prevalence survey of health care–associated infections. N. Engl. J. Med..

[3] Kumamoto C.A. (2008). Molecular mechanisms of mechanosensing and their roles in fungal contact sensing. Nat. Rev. Microbiol..

[4] Cavalheiro M., Teixeira M.C. (2018). *Candida* Biofilms: Threats, Challenges, and Promising Strategies. Front. Med..

[5] Gow N.A., Brown A.J., Odds F.C. (2002). Fungal morphogenesis and host invasion. Curr. Opin. Microbiol..

[6] Sterzenbach T., Helbig R., Hannig C., Hannig M. (2020). Bioadhesion in the oral cavity and approaches for biofilm management by surface modifications. Clin. Oral Investig..

[7] Liu Y.S., Lee O.K. (2014). In Search of the Pivot Point of Mechanotransduction: Mechanosensing of Stem Cells. Cell Transplant..

[8] Viela F., Granados D., Ayuso-Sacido A., Rodríguez I. (2016). Biomechanical Cell Regulation by High Aspect Ratio Nanoimprinted Pillars. Adv. Funct. Mater..

[9] Le Saux G., Bar-Hanin N., Edri A., Hadad U., Porgador A., Schvartzman M. (2019). Nanoscale Mechanosensing of Natural Killer Cells is Revealed by Antigen-Functionalized Nanowires. Adv. Mater..

[10] Fu J., Liu X., Tan L., Cui Z., Liang Y., Li Z., Zhu S., Zheng Y., Kwok Yeung K.W., Chu P.K. (2020). Modulation of the Mechanosensing of Mesenchymal Stem Cells by Laser-Induced Patterning for the Acceleration of Tissue Reconstruction through the Wnt/β-Catenin Signaling Pathway Activation. Acta Biomater..

[11] Zhou C., Zhang D., Du W., Zou J., Li X., Xie J. (2020). Substrate Mechanics Dictate Cell-Cell Communication by Gap Junctions in Stem Cells from Human Apical Papilla. Acta Biomater..

[12] Dalby M.J. (2005). Topographically induced direct cell mechanotransduction. Med. Eng. Phys..

[13] Salou L., Hoornaert A., Louarn G., Layrolle P. (2015). Enhanced osseointegration of titanium implants with nanostructured surfaces: An experimental study in rabbits. Acta Biomater..

[14] Wandiyanto J.V., Linklater D., Tharushi Perera P.G., Orlowska A., Truong V.K., Thissen H., Ghanaati S., Baulin V., Crawford R.J., Juodkazis S. (2018). Pheochromocytoma (PC12) Cell Response on Mechanobactericidal Titanium Surfaces. Materials.

[15] Senevirathne S.W.M.A.I., Hasan J., Mathew A., Woodruff M., Yarlagadda P.K.D.V. (2021). Bactericidal efficiency of micro- and nanostructured surfaces: A critical perspective. RSC Adv..

[16] Takai E., Costa K.D., Shaheen A., Hung C.T., Guo X.E. (2005). Osteoblast Elastic Modulus Measured by Atomic Force Microscopy Is Substrate Dependent. Ann. Biomed. Eng..

[17] Abagnale G., Steger M., Nguyen V.H., Hersch N., Sechi A., Joussen S., Denecke B., Merkel R., Hoffmann B., Dreser A. (2015). Surface topography enhances differentiation of mesenchymal stem cells towards osteogenic and adipogenic lineages. Biomaterials.

[18] Ivanova E.P., Hasan J., Webb H.K., Gervinskas G., Juodkazis S., Truong V.K., Wu A.H.F., Lamb R.N., Baulin V.A., Watson G.S. (2013). Bactericidal activity of black silicon. Nat. Commun..

[19] Aburto-Medina A., Le P.H., MacLaughlin S., Ivanova E. (2021). Diversity of experimental designs for the fabrication of antifungal surfaces for the built environment. Appl. Microbiol. Biotechnol..

[20] Razvag Y., Neve-Oz Y., Sherman E., Reches M. (2019). Nanoscale Topography-Rigidity Correlation at the Surface of T Cells. ACS Nano.

[21] Chen Z., Zhu Y., Xu D., Alam M.M., Shui L., Chen H. (2020). Cell Elasticity Measurement Using a Microfluidic Device with Real-Time Pressure Feedback. Lab Chip.

[22] Zhang X.C., Guo Y., Liu X., Chen X.G., Wu Q., Chen G.Q. (2018). Engineering Cell Wall Synthesis Mechanism for Enhanced PHB Accumulation in *E. coli*. Metab. Eng..

[23] Asgari M., Brulé V., Western T.L., Pasini D. (2020). Nano-Indentation Reveals a Potential Role for Gradients of Cell Wall Stiffness in Directional Movement of The Resurrection Plant *Selaginella lepidophylla*. Sci. Rep..

[24] Heinisch J.J., Rodicio R., König H., Unden G., Fröhlich J. (2009). Physical and Chemical Stress Factors in Yeast. Biology of Microorganisms on Grapes, in Must and in Wine.

[25] Nguyen D.H.K., Loebbe C., Linklater D.P., Xu X., Vrancken N., Katkus T., Juodkazis S., Maclaughlin S., Baulin V., Crawford R.J. (2019). The idiosyncratic self-cleaning cycle of bacteria on regularly arrayed mechano-bactericidal nanostructures. Nanoscale.

[26] Hao Y., Cheng S., Tanaka Y., Hosokawa Y., Yalikun Y., Li M. (2020). Mechanical properties of single cells: Measurement methods and applications. Biotechnol. Adv..

[27] Le P.H., Nguyen D.H.K., Aburto-Medina A., Linklater D.P., Crawford R.J., MacLaughlin S., Ivanova E.P. (2020). Nanoscale Surface Roughness Influences *Candida albicans* Biofilm Formation. ACS Appl. Bio Mater..

[28] Ivanova E.P., Truong V.K., Wang J.Y., Berndt C.C., Jones R.T., Yusuf I.I., Peake I., Schmidt H.W., Fluke C., Barnes D. (2010). Impact of nanoscale roughness of titanium thin film surfaces on bacterial retention. Langmuir.

[29] Nečas D., Klapetek P. (2012). Gwyddion: An open-source software for SPM data analysis. Open Phys..

[30] Abràmoff M.D., Magalhães P.J., Ram S.J. (2004). Image processing with ImageJ. Biophoton. Int..

[31] Galdiero E., de Alteriis E., De Natale A., D’Alterio A., Siciliano A., Guida M., Lombardi L., Falanga A., Galdiero S. (2020). Eradication of *Candida albicans* persister cell biofilm by the membranotropic peptide gH625. Sci. Rep..

[32] Hassan A.N., Frank J.F., Qvist K.B. (2002). Direct observation of bacterial exopolysaccharides in dairy products using confocal scanning laser microscopy. J. Dairy Sci..

[33] Adya A.K., Canetta E., Walker G.M. (2006). Atomic force microscopic study of the influence of physical stresses on *Saccharomyces cerevisiae* and *Schizosaccharomyces pombe*. FEMS Yeast Res..

[34] Lin D.C., Dimitriadis E.K., Horkay F. (2007). Robust Strategies for Automated AFM Force Curve Analysis—II: Adhesion-Influenced Indentation of Soft, Elastic Materials. J. Biomech. Eng..

[35] Schindelin J., Arganda-Carreras I., Frise E., Kaynig V., Longair M., Pietzsch T., Preibisch S., Rueden C., Saalfeld S., Schmid B. (2012). Fiji: An Open-Source Platform for Biological-Image Analysis. Nat. Methods.

[36] Chen H., Zhou X., Ren B., Cheng L. (2020). The regulation of hyphae growth in *Candida albicans*. Virulence.

[37] Nguyen D.H.K., Wang J., Sbarski I., Juodkazis S., Crawford R.J., Ivanova E.P. (2019). Influence of Amorphous, Carbon-Derived Wrinkled Surface Topologies on the Colonization of *Pseudomonas aeruginosa* Bacteria. Adv. Mater. Interfaces.

[38] Formosa C., Schiavone M., Martin-Yken H., François J.M., Duval R.E., Dague E. (2013). Nanoscale effects of caspofungin against two yeast species, *Saccharomyces cerevisiae* and *Candida albicans*. Antimicrob. Agents Chemother..

[39] Kheur S., Singh N., Bodas D., Rauch J.-Y., Jambhekar S., Kheur M., Rajwade J. (2017). Nanoscale silver depositions inhibit microbial colonization and improve biocompatibility of titanium abutments. Colloids Surf. B Biointerfaces.

[40] Bhat S.V., Sultana T., Körnig A., McGrath S., Shahina Z., Dahms T.E.S. (2018). Correlative Atomic Force Microscopy Quantitative Imaging-Laser Scanning Confocal Microscopy Quantifies the Impact of Stressors on Live Cells in Real-Time. Sci. Rep..

[41] Khoury Z.H., Vila T., Puthran T.R., Sultan A.S., Montelongo-Jauregui D., Melo M.A.S., Jabra-Rizk M.A. (2020). The Role of *Candida albicans* Secreted Polysaccharides in Augmenting *Streptococcus* mutans Adherence and Mixed Biofilm Formation: In vitro and in vivo Studies. Front. Microbiol..

[42] Akashi T., Kanbe T., Tanaka K. (1994). The role of the cytoskeleton in the polarized growth of the germ tube in *Candida albicans*. Microbiology.

[43] Walther A., Wendland J. (2004). Polarized hyphal growth in *Candida albicans* requires the Wiskott-Aldrich Syndrome protein homolog Wal1p. Eukaryot. Cell.

[44] Pruyne D., Legesse-Miller A., Gao L., Dong Y., Bretscher A. (2004). Mechanisms of polarized growth and organelle segregation in yeast. Annu. Rev. Cell Dev. Biol..

[45] Sheikh-Hamad D., Gustin M.C. (2004). MAP kinases and the adaptive response to hypertonicity: Functional preservation from yeast to mammals. Am. J. Physiol. Ren. Physiol..

[46] Oberholzer U., Nantel A., Berman J., Whiteway M. (2006). Transcript profiles of *Candida albicans* cortical actin patch mutants reflect their cellular defects: Contribution of the Hog1p and Mkc1p signaling pathways. Eukaryot. Cell.

[47] Garcia-Rubio R., de Oliveira H.C., Rivera J., Trevijano-Contador N. (2020). The Fungal Cell Wall: *Candida*, *Cryptococcus*, and *Aspergillus* Species. Front. Microbiol..

[48] Schiavone M., Formosa-Dague C., Elsztein C., Teste M.A., Martin-Yken H., De Morais M.A., Dague E., François J.M. (2016). Evidence for a Role for the Plasma Membrane in the Nanomechanical Properties of the Cell Wall as Revealed by an Atomic Force Microscopy Study of the Response of *Saccharomyces cerevisiae* to Ethanol Stress. Appl. Environ. Microbiol..

[49] Quilès F., Accoceberry I., Couzigou C., Francius G., Noël T., El-Kirat-Chatel S. (2017). AFM Combined to ATR-FTIR Reveals *Candida* Cell Wall Changes under Caspofungin Treatment. Nanoscale.

[50] Gonçalves S., Silva P.M., Felício M.R., de Medeiros L.N., Kurtenbach E., Santos N.C. (2017). Psd1 Effects on *Candida albicans* Planktonic Cells and Biofilms. Front. Cell. Infect. Microbiol..

[51] Ma S., Ge W., Yan Y., Huang X., Ma L., Li C., Yu S., Chen C. (2017). Effects of *Streptococcus sanguinis* Bacteriocin on Deformation, Adhesion Ability, and Young’s Modulus of *Candida albicans*. Biomed. Res. Int..

[52] Çolak A., Ikeh M.A.C., Nobile C.J., Baykara M.Z. (2020). In Situ Imaging of *Candida albicans* Hyphal Growth via Atomic Force Microscopy. mSphere.

[53] Ene I.V., Adya A.K., Wehmeier S., Brand A.C., Maccallum D.M., Gow N.A.R., Brown A.J.P. (2012). Host Carbon Sources Modulate Cell Wall Architecture, Drug Resistance and Virulence in A Fungal Pathogen. Cell. Microbiol..

[54] Ene I.V., Walker L.A., Schiavone M., Lee K.K., Martin-Yken H., Dague E., Gow N.A.R., Munro C.A., Brown A.J.P. (2015). Cell Wall Remodeling Enzymes Modulate Fungal Cell Wall Elasticity and Osmotic Stress Resistance. mBio.

[55] Yang L., Gao Q., Ge L., Zhou Q., Warszawik E.M., Bron R., Lai K.W.C., Van Rijn P. (2020). Topography Induced Stiffness Alteration of Stem Cells Influences Osteogenic Differentiation. Biomater. Sci..

[56] Alalwan H., Nile C.J., Rajendran R., McKerlie R., Reynolds P., Gadegaard N., Ramage G. (2018). Nanoimprinting of biomedical polymers reduces candidal physical adhesion. Nanomed. Nanotechnol. Biol. Med..

[57] Pessoa R.S., dos Santos V.P., Cardoso S.B., Doria A.C.O.C., Figueira F.R., Rodrigues B.V.M., Testoni G.E., Fraga M.A., Marciano F.R., Lobo A.O. (2017). TiO_2_ coatings via atomic layer deposition on polyurethane and polydimethylsiloxane substrates: Properties and effects on *C. albicans* growth and inactivation process. Appl. Surf. Sci..

[58] Nowlin K., Boseman A., Covell A., LaJeunesse D. (2015). Adhesion-dependent rupturing of *Saccharomyces cerevisiae* on biological antimicrobial nanostructured surfaces. J. R. Soc. Interface.

[59] Kollu N.V., LaJeunesse D.R. (2021). Cell Rupture and Morphogenesis Control of the Dimorphic Yeast *Candida albicans* by Nanostructured Surfaces. ACS Omega.

[60] Chaffin W.L., Lopez-Ribot J.L., Casanova M., Gozalbo D., Martinez J.P. (1998). Cell wall and secreted proteins of *Candida albicans*: Identification, function, and expression. Microbiol. Mol. Biol. Rev..

