# Supplementary material for: Surface Architecture Influences the Rigidity of Candida albicans Cells
Source: Nanomaterials (Basel). 2022 Feb 7;12(3):567. doi: 10.3390/nano12030567 (PMC8840568; doi:10.3390/nano12030567)
Supplement: Supplementary file 1 [file nanomaterials-12-00567-s001.zip › nanomaterials-1536593-supplementary.pdf]

Supplementary information

Surface architecture influences the rigidity of *Candida albicans* cells

Phuc H. Le[a,b†], Duy H. K. Nguyen[a†], Arturo Aburto Medina[a,b], Denver P. Linklater[a], Christian Loebbe[c], Russell J. Crawford[a], Shane Maclaughlin[d], and Elena P. Ivanova[a*]

[a] College of STEM, School of Science, RMIT University, Melbourne, VIC 3000, Australia

[b] ARC Research Hub for Australian Steel Manufacturing, Australia

[c] Scitech P/L, Preston, VIC, 3072, Australia

[d] BlueScope Steel Ltd, Port Kembla, NSW 2505, Australia

[†] Equal contribution

*Corresponding author: elena.ivanova@rmit.edu.au

RMIT Classification: Trusted

Table S1. Elemental composition and wettability of Ti, pTi and glass surfaces

**Surfaces**	**Atomic fractions of element (Wt.%)**[a]									
	Ti	O	Na	Mg	Al	Si	K	Ca	W	Au
**npTi**	94.4	5.6	<dl[b]	<dl	<dl	<dl	<dl	<dl	<dl	<dl
**pTi**	96.9	3.1	<dl	<dl	<dl	<dl	<dl	<dl	<dl	<dl
**glass**	<dl	38.6	8.7	2.6	0.4	37.6	0.4	7.8	<dl	3.8

	**npTi**	**pTi**	**glass**
**Contact angle θ (°)**[c]	$58.8 \pm 4.8^{*}$	64.9 ± 0.9	$5.3 \pm 2.0^{*}$

[a] Atomic fractions of elements detected by using EDS

[b] <dl means below the detection limit

[c] Water contact angle was measured in static conditions, n = 5

[*] The asterisk indicates values of statistically significant difference (in row) compared to pTi surfaces, $p < 0.05$

RMIT Classification: Trusted

Table S2. The size of *C. albicans* cells from suspension and attached on npTi, pTi and glass

	**Suspension**	**npTi**	**pTi**	**Glass**
Cell size[a] (μm)	5.83 ± 0.67	3.83 ± 0.63*	3.56 ± 0.67*	3.78 ± 0.57*

[a] Cell size was measured using SEM micrographs, n = 35

[*] The asterisk indicates values of statistically significant difference (in row) compared to suspension, $p < 0.05$

RMIT Classification: Trusted

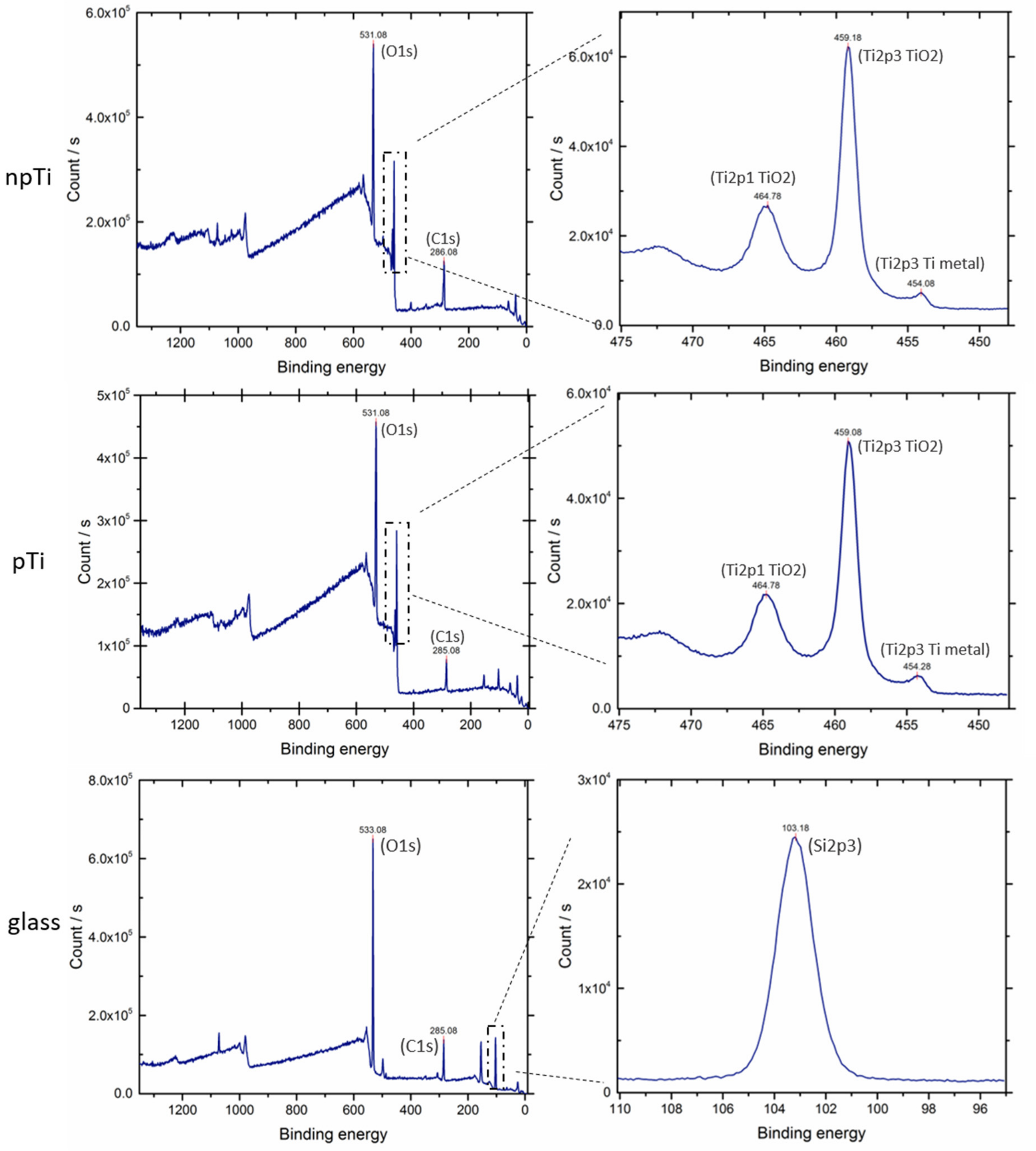

Figure S1. The chemical composition of npTi, pTi and glass surfaces. XPS-spectra of npTi, pTi and glass surfaces (left) and correlating narrow scan spectra of Ti2p and Si2p positions (right). There were two dominant doublet peaks, Ti2p3 (binding energy = 496 eV) and Ti2p1 (binding energy = 465 eV), which can be referred to TiO_2. The peak at 454 eV was Ti2p3 which can be attributed to metallic Ti. There was not a significant change in the chemical composition of pTi after the polishing process. In terms of glass surfaces, three dominant peaks of carbon (C1s, BE = 285 eV), silicon (Si2p3, BE = 103 eV) and oxygen (O1s, BE = 532 eV) were determined.

RMIT Classification: Trusted

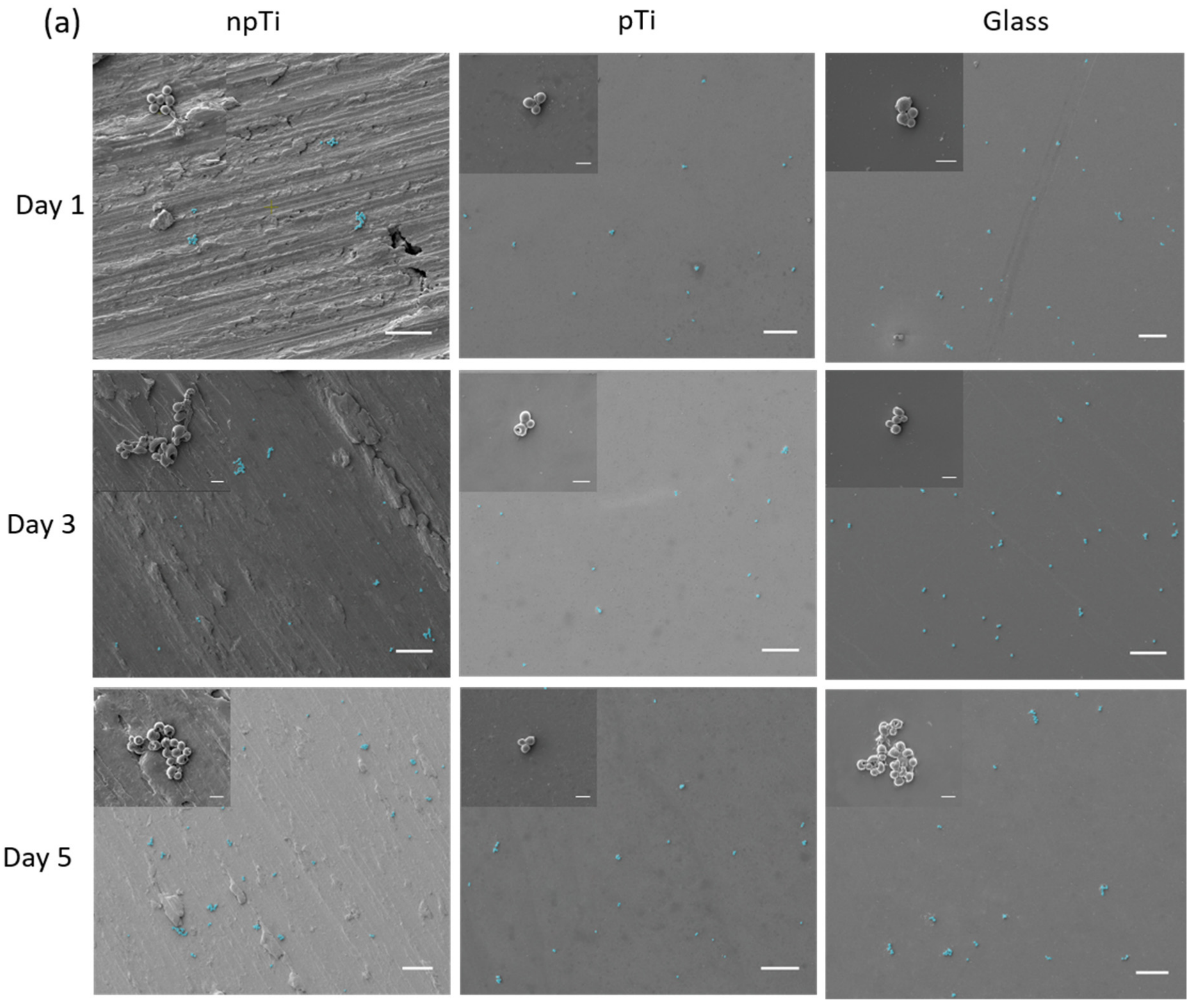

Figure S2. Representative SEM micrographs of *C. albicans* cells attached on npTi, pTi and glass. Attached cells were highlighted in cyan color. Scale bars are 50 μm and 5 μm for the overview and inset micrographs, respectively.

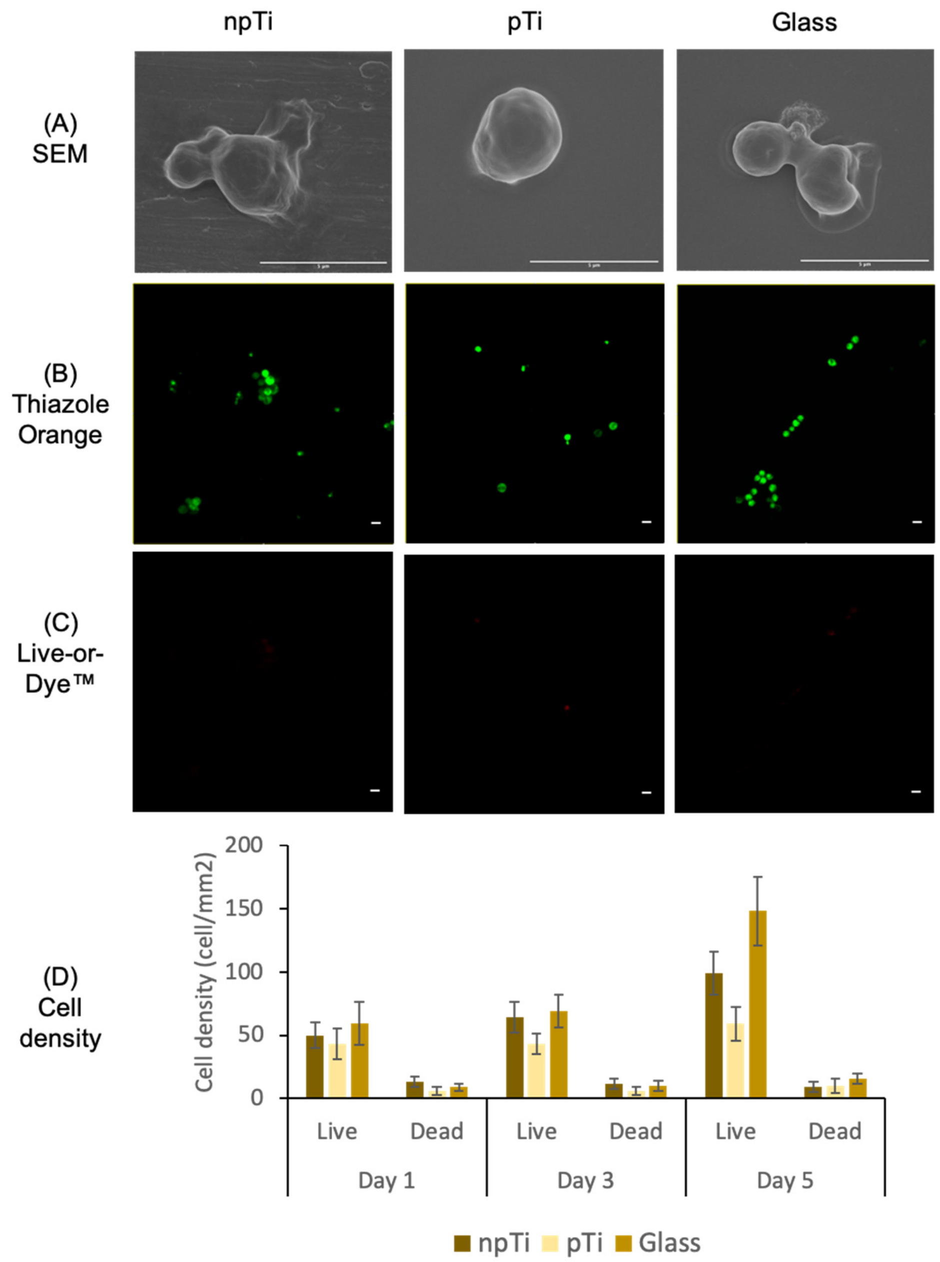

Figure S3. The morphology, viability, and attachment patterns of *C. albicans* cells on npTi, pTi and glass surfaces. (A) Representative SEM micrograph showing the morphology of attached *C. albicans* cells on the three studied surfaces. On npTi and glass surfaces, EPS was developed on attached cells. (B) Live cells (green colour) were stained by Thiazole Orange. (C) Dead cells (red colour) were stained with Live-or-Dye™. (D) Quantification of the total live and dead *C. albicans* cells on the different surfaces over 5-day incubation. The numbers of live and dead cells were calculated using the CLSM images (n = 10). The scanning area of the CLSM was 135 × 135 μm^2.

RMIT Classification: Trusted

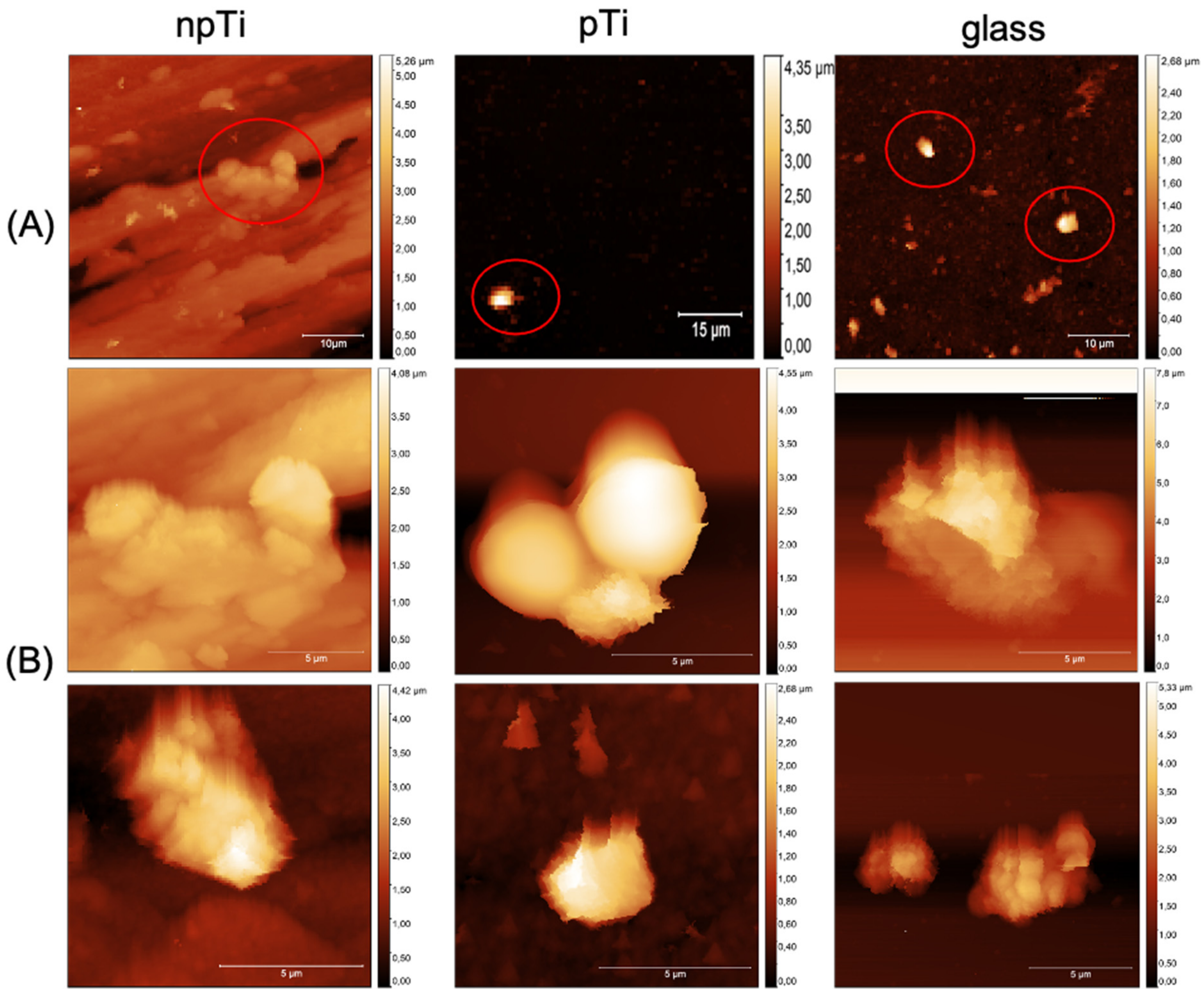

Figure S4. AFM micrographs showing the shape and orientation of cell attachment on npTi, pTi and glass. (A) Overview of attached cells on surfaces scanned in low resolution mode (128 × 128 pixels). The red circles indicate *C. albicans* cells. (B) 2D AFM micrographs demonstrated the morphology of attached *C. albicans* cells on the studied surfaces which possessed different surface nanoarchitectures.

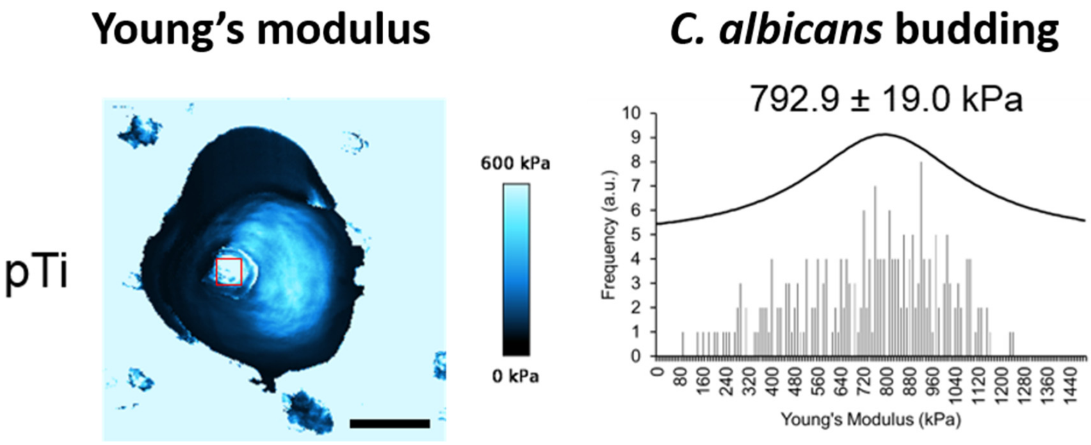

Figure S5. The mechanical properties of *C. albicans* budding. Representative Young's modulus of the budding of *C. albicans* cells attached on npTi, glass and pTi surfaces after a 3-day incubation period. The elasticity of *C. albicans* budding was obtained by using Hertz/Seddon model for soft materials on 65,536 AFM force curves. The dark and bright blue colour in elasticity maps represented soft and hard matters, respectively. Young's modulus data highlighted by red square were extracted and constructed into a distribution. These histograms were fitted with Gaussian function to observe a nominal Young's modulus value. Scale bar is 2 μm.

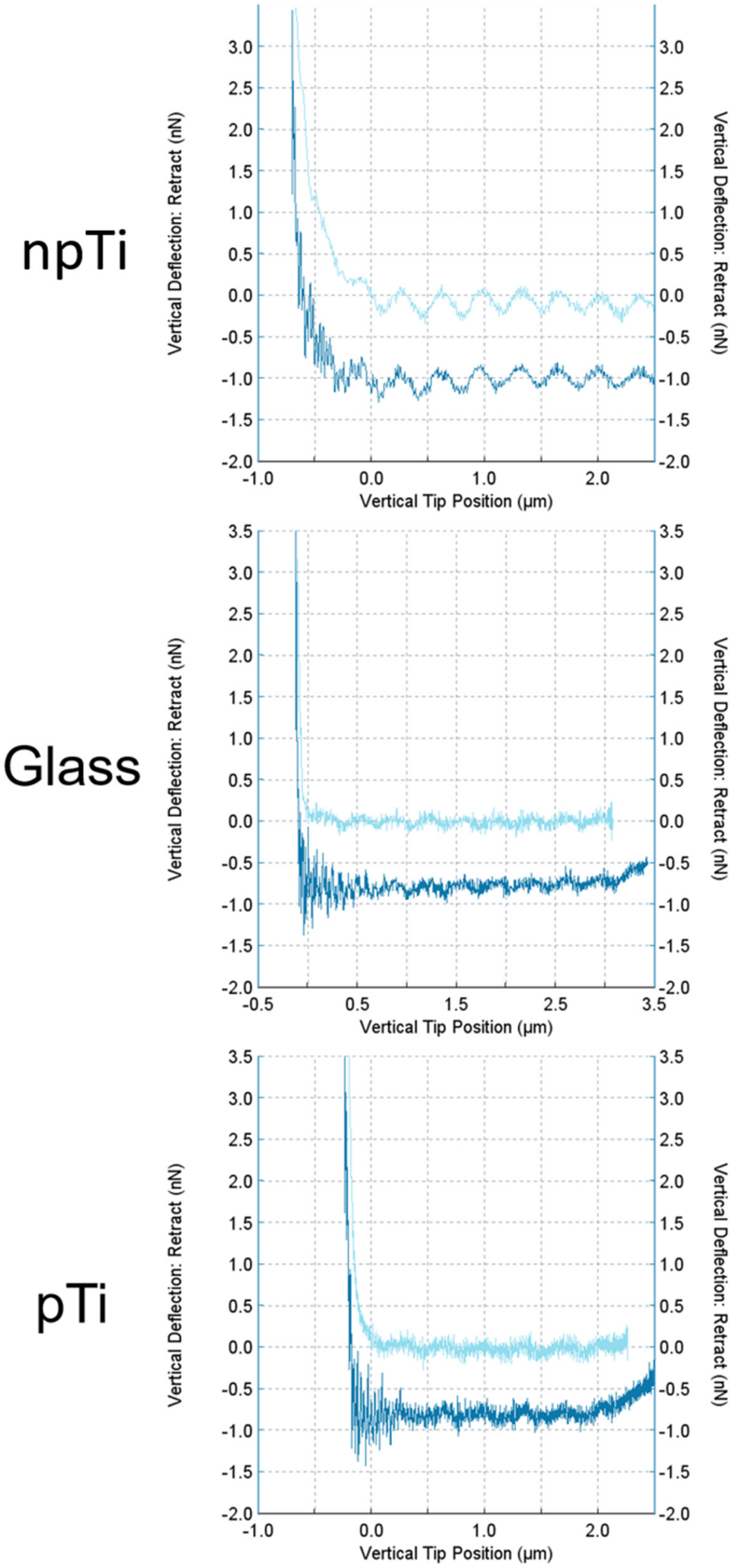

Figure S6. Representative force-distance curves in QI data of *C. albicans* cells attached on npTi, glass and pTi surfaces after a 3-day incubation period.
